# Period-2: a tumor suppressor gene in breast cancer

**DOI:** 10.1186/1740-3391-6-4

**Published:** 2008-03-11

**Authors:** Shulin Xiang, Seth B Coffelt, Lulu Mao, Lin Yuan, Qi Cheng, Steven M Hill

**Affiliations:** 1 Department of Structural and Cellular Biology, Tulane University Health Sciences Center, New Orleans, LA 70112, USA; 2 Department of Microbiology and Immunology, Tulane University Health Sciences Center, New Orleans, LA 70112, USA; 3 Tulane Cancer Center, Tulane University Health Sciences Center, New Orleans, LA 70112, USA

## Abstract

Previous reports have suggested that the ablation of the *Period 2 *gene (*Per 2*) leads to enhanced development of lymphoma and leukemia in mice. Employing immunoblot analyses, we have demonstrated that *PER 2 *is endogenously expressed in human breast epithelial cell lines but is not expressed or is expressed at significantly reduced level in human breast cancer cell lines. Expression of *PER 2 *in MCF-7 breast cancer cells significantly inhibited the growth of MCF-7 human breast cancer cells, and, when *PER 2 *was co-expressed with the Crytochrome 2 (*Cry 2*) gene, an even greater growth-inhibitory effect was observed. The inhibitory effect of *PER 2 *on breast cancer cells was also demonstrated by its suppression of the anchorage-independent growth of MCF-7 cells as evidenced by the reduced number and size of colonies. A corresponding blockade of MCF-7 cells in the G1 phase of the cell cycle was also observed in response to the expression of *PER 2 *alone or in combination with *CRY 2*. Expression of *PER 2 *also induced apoptosis of MCF-7 breast cancer cells as demonstrated by an increase in PARP [poly (ADP-ribose) polymerase] cleavage. Finally, our studies demonstrate that *PER 2 *expression in MCF-7 breast cancer cells is associated with a significant decrease in the expression of cyclin D1 and an up-regulation of p53 levels.

## Background

In mammals, most body functions follow a rhythmic pattern adjusted to a 24 h period (circadian rhythm), which is controlled by the circadian timing system [1,2]. Circadian rhythmicity is an evolutionarily conserved property that regulates numerous functions in the human body including sleep and wakefulness, body temperature, blood pressure, hormone production, digestive secretion, and immune activity [3]. The circadian timing system comprises peripheral oscillators located in most tissues of the body and a central rhythm generator located in the suprachiasmatic nucleus (SCN) of the hypothalamus [4]. The SCN pacemaker consists of multiple, autonomous single cell circadian oscillators, which are synchronized to fire rhythmically, generating a coordinated, rhythmic output in intact animals [5,6].

The cellular mechanism of circadian rhythmicity involves the regulation of three *Period *genes (*Per 1–3*) and two *Chrytochrome *genes (*Cry1 *and *2*) [4]. Currently, it is thought that transcription of *Per *and *Cry *genes is driven by accumulating CLOCK:BMAL1 heterodimers, which in turn bind to consensus E-box elements [7-10]. Subsequently, complexes of *PER *2 and *CRY *2 proteins enter the nucleus, where they shut off CLOCK-mediated transcription. At the same time, *PER 2 *up-regulates the levels of BMAL1 mRNA leading to the formation of CLOCK:BMAL1 heterodimers, which drive *Per 2 *and *Cry 2 *transcription and restart the cycle [11,12]. MOP4 (member of the PAS superfamily 4), also named NPAS2, shares high homology with CLOCK [13] and like CLOCK forms a heterodimer with BMAL1, promoting E-box activation of genes such as *Per1 *and vasopressin and is negatively regulated by *CRY 1 *and *2 *[11]. In adult animals, oscillatory expression of CLOCK genes has been demonstrated in the SCN and in several peripheral tissues. Interacting positive and negative transcriptional-translational feedback loops drive circadian oscillators in both *Drosphila *and mammals. Furthermore, immortalized rat fibroblasts harbor a clock that can measure time with astonishing precision [14]. The SCN clock is thought to synchronize such peripheral clocks via both neural and hormonal signals.

For several years now, it has been known that disruption of circadian rhythm increases the rate of tumorigenesis [15,16], but until recently no molecular evidence was available to explain this phenomenon. Breast cancer is especially susceptible to circadian alterations due to the fact that it is an endocrine responsive neoplasm, and many hormones known to influence the development and growth of the breast and breast cancer exhibit diurnal rhythms of synthesis and secretion [17-21].

Numerous epidemiological studies have implied a role for the circadian clock in breast cancer development. A Danish study investigating 30- to 54-year old women reported that women who work predominantly night shifts, and thus are exposed to light at night, show a significantly increased risk of breast cancer [18]. The breast cancer risk increases significantly with the number of years and hours that individuals spend working at night [18,22]. Similar results are described by Schernhammer et al. [23] who examined 78,562 nurses that often alternate between day and night shifts. A meta analysis of all of these studies demonstrate that circadian rhythm interruption significantly increases an individual's risk for the development of breast cancer. Based on our work [24,25] and that of others that melatonin, a photoperiodic hormone whose expression is repressed by light, inhibits the growth and development of breast cancer, we believe that pro-tumorigenic effects of light are mediated through melatonin and its effect on the clock an circadian rhythms.

In tumor-bearing animals and cancer patients, circadian disruption not only increases the risk of tumor development, but also accelerates cancer progression and is associated with poor prognosis and outcome. Filipski et al. [26] demonstrated that complete ablation of the SCN in mice results in loss of circadian rhythm as well as a two- to three-fold increase in malignant growth when compared to controls, leading to significant reductions in survival time. As well, carcinoma- or sarcoma-bearing rats show an increase in tumor growth and a reduction in survival time when subjected to alternating photoperiods [27]. Other studies that have disrupted circadian rhythms in mice by targeted mutations of the core clock genes engendering the molecular clock not only in the suprachiasmatic nuclei but in all peripheral organs have shown effects on disruption of cell growth and spontaneous and ionizing radiation-induced tumor incidence [28].

At present, the mechanism by which the circadian clock affects tumor growth is not fully understood. Recently, the circadian clock gene *Per 2*, which helps to synchronize mammalian organisms with environmental photic cues, has been reported to function as a tumor suppressor gene. Fu et al. [28] observed the development of spontaneous lymphomas and teratomas in *Per 2 *knockout mice at only six months of age. Thirty per cent of the mutant mice died before the age of 16 months. Disruption of the *Per 2 *gene in mice abolishes the response of all core circadian genes to gamma radiation whereas in wild-type mice the clock genes are induced rapidly, suggesting they may be involved in DNA damage response. Furthermore, a number of cell cycle and checkpoint proteins were deregulated in these *Per 2 *mutant mice including cyclin D1, cyclin A, mdm-2, gadd45α, and c-myc. The studies cited above demonstrate the importance of circadian clock genes, in particular *Per 2*, as regulators of the cell cycle and, therefore, cancer progression. Based on the role of *PER 2 *in cancer development and the clear epidemiologic connection between circadian disruption (light at night) and the risk of breast cancer development in women, we hypothesize that the clock gene, Per 2, is expressed in normal human mammary epithelium and at a reduced level in breast cancer cells leading to an alteration in the cell cycle, cell growth, and cell survival.

## Materials and methods

### Human breast cancer and breast epithelial cell lines

The human breast cancer cell line MDA-MB-231 and the immortalized MCF-10A human breast epithelial cell line were purchased from American Tissue Type Culture Collection (Rockville, MD). The MCF-7 breast tumor cell line was obtained from the laboratory of the late William L. McGuire (San Antonio, TX),. The T47D breast tumor cell line was kindly provided by Dr. I. Keydar (Tel Aviv University, Israel). The human mammary epithelial cell line hTERT-HME1 was provided by Dr. Matthew Burow (Tulane University). All cells except hTERT-HME1 cells were routinely maintained in RPMI 1640 medium supplemented with 10% fetal bovine serum (FBS) [Gibco BRL, Grand Island, NY], 2 mM glutamine, 50 mM MEM non-essential amino acids, 1 mM sodium pyruvate, 10 mM basal medium eagle (BME), 100 U/ml penicillin, and 100 mg/ml streptomycin. The hTERT-HME1 cells were grown in serum-free MEBM medium (Cambrex Bio Science Walkersville Inc., Walkersville, MD) supplemented with 52 μg/ml bovine pituitary extract. All cells were incubated in a humidified atmosphere of 5% CO_2 _and 95% air at a constant temperature of 37ºC.

### Antibodies, plasmids and recombinant DNAs

The mouse antibody against human *PER 2 *and the rabbit anti-goat IgG and horseradish peroxidase-conjugated secondary antibodies were purchased from Santa Cruz Biotechnology (Santa Cruz, CA). Mouse antibodies to PARP [poly (ADP-ribose) polymerase], P53, Cyclin D1 and β-actin were purchased from Sigma Chemical Co. (St. Louis, MO). Recombinant DNAs for the human *Per 2 *and *Cry 2 *genes were kindly provided by David Virshup (Salt Lake City, UT) and Charles Weitz (Harvard Medical School, Boston, MA), respectively. Plasmids used in these studies consisted of the pcDNA 3.1 vector and the GFP vector pTracer™-CMV2, which were purchased from Invitrogen (Carlsbad, CA).

### Cell culture and transfection

MCF-7 breast cancer cells were maintained in RPMI 1640 supplemented with antibiotics (BioWhitaker, Walkersville, MD, USA) and 10% FBS in a humidified atmosphere of 5% CO_2 _at 37ºC. MCF-7 breast cancer cells were then plated in 6-well plates at a density of 0.5 × 10^5 ^cells/well (for protein isolation) or 2 × 10^4 ^cells/well (for growth studies) in the same media and transfected on the following day. The cells were transiently transfected with the pcDNA 3.1 or pCS2+MT empty vectors or the same plasmids containing the human *Per 2 *or *Cry 2 *genes (400 ng/well of plasmid or plasmid plus cDNA) using the FuGENE 6 transfection reagent (Roche Diagnostics, Indianapolis, IN) for 6–8 h in serum-free medium. Following transfection the cells were re-fed with medium supplemented with 10% FBS.

### Protein isolation and Western blot analysis

MCF-7 cells were transiently transfected with empty vectors, *Per 2 *cDNA or both *Per 2 *and *Cry 2 *cDNA as described above. Forty eight hours post transfection, total cellular protein was extracted by suspending the cell pellets in lysis buffer (50 mM Tris, pH 8.0, 150 mM NaCl, 0.1% SDS, 0.5% sodium deoxycholate, 0.1% Triton X-100, 1 mM PMSF, 0.02% sodium azide, 1 mg/ml aprotinin and 1 mg/ml leupeptin) for 30 min at 4ºC and then centrifuging at 10,000 × *g *for 10 min. Supernatants were collected as total cellular protein and assayed for protein concentration using the Bio-Rad protein assay system (BioRad, Hercules, CA).

One hundred micrograms of total cellular protein per sample was electrophoretically separated on a 10% sodium dodecyl sulfate (SDS) polyacrylamide gel and transferred onto a nylon membrane (Amersham Life Science) by electroblotting. The membranes were blocked by incubation for 90 min at room temperature with 5% (w/v) nonfat dry milk in TBST (150 mM NaCl, 10 mM Tris-HCl, 0.1% TWEEN), then incubated with antibodies directed against the *PER 2*, PARP, p53, cyclin D1 or β-actin (1:250 dilution) overnight at 4ºC, washed with TBST, and incubated for 1 h at room temperature with horseradish peroxidase-conjugated rabbit-anti-mouse IgG or goat-anti-rabbit IgG (1:10,000 dilution; Amersham Life Science) secondary antibody. Immunoreactive proteins were visualized using the enhanced chemiluminescence system (ECL; Amersham Life Science). Membranes were exposed for 3 to 30 min to Kodak X-OMAT AR film. Expression values of proteins were normalized to the β-actin (loading control) and quantitated by scanning densitometry using a Bio-Rad GS-700 imaging densitometer.

### Growth studies

MCF-7 breast cancer cells were plated in 6-well plates at a density of 2 × 10^4 ^cells/well in media supplemented with 10% FBS and transfected on the following day. The cells were transiently transfected with the pcDNA 3.1 vector or/and the pCS2+MT empty vectors or the same plasmids containing the human *Per 2 *or *Cry 2 *genes as described above. On each day following transfection cells were harvested in phosphate-buffered saline (PBS)/EDTA solution containing 0.1% trypsin and counted on a heamocytometer every day for four days using the Trypan blue dye exclusion method.

### Soft agar clonogenic assay

MCF-7 cell were transfected with pTracer™-CMV2 or pTracer™-CMV2-hPer 2 for 18 h, pTracer™-CMV2-positive or pTracer™-CMV2-hPer 2-positive cells were sorted with Becton Dickenson Flow Activated Cell Sorter (FACS) Aria. Two thousand five hundred cells per well were seeded in 12-well plates (3.8 cm^2^) in culture medium containing 0.35% low-melting agarose over a 0.7% agarose base layer and incubated for 12 days at 37ºC in a humidified 5% CO_2 _atmosphere in RPMI 1640 media supplemented with 10% FBS. Colonies larger than 100 μm in diameter were counted under a dissecting microscope. Each cell sample was seeded in triplicate and soft agar assays were repeated three separate times.

### Cell cycle studies

MCF-7 cells were transiently transfected with empty vectors, *Per 2 *cDNA or both *Per 2 *and *Cry 2 *cDNA. After three days of expression, cells were harvested with PBS/EDTA and the cells pelleted at 1000 × g for 5 minutes. Cell pellets were washed once in cold PBS and resuspended in 500 μl of cold PBS with 0.1% glucose. Five milliliters of cold 70% ethanol, were added to the cell suspension and the cells were kept at 4ºC for 30 min. to 12 h. Cells were pelleted at 1000 × g for 5 minutes, the supernatant removed and the cells washed with 5 ml of cold PBS. The cells were then resuspended in 300 μl of propidium iodide (PI)/Triton X-100 staining solution with RNase A, and incubated for 30 min at 37ºC. Samples were then examined for cell cycle phase by flow cytometry (BDLSR11; Becton Dickinson, San Jose, CA).

## Results

### Expression of PER 2 in human breast epithelial and breast cancer cell lines

**Figure 1:**
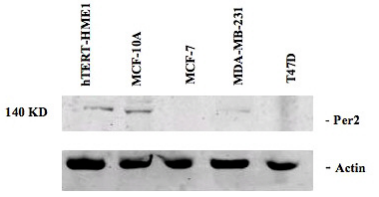
**Expression of *PER 2 *in human breast epithelial and breast cancer cell lines**. Total cellular protein was isolated from two immortalized human breast epithelial cell lines (HME-tert and MCF-10A), two ERα-positive human breast tumor cell lines (MCF-7 and T47D lines) and one ERα-negative human breast tumor cell lines (MDA-MB-231 line). One hundred micrograms of total cellular protein from each cell line was separated by 10% polyacrylamide gel electrophoresis and subjected to Western blot analysis using an antibody directed against the human *PER 2 *protein.

Total cellular protein was isolated from two immortalized human breast epithelial cell lines (HME-tert and MCF-10A), two ERα-positive human breast tumor cell lines (MCF-7 and T47D lines) and one ERα-negative human breast tumor cell lines (MDA-MB-231), and was examined by Western blot analysis using an antibody directed against the human *PER 2 *protein (Figure 1). Expression of *PER 2 *was evident in the two breast epithelial cell lines, but was not observed in MCF-7 and T47D cell lines. It was present in the MDA-MB-231 cell line, but at a level greatly reduced compare to the breast epithelial cell lines.

### Effect of PER 2 and CRY 2 on the growth of MCF-7 cells

**Figure 2:**
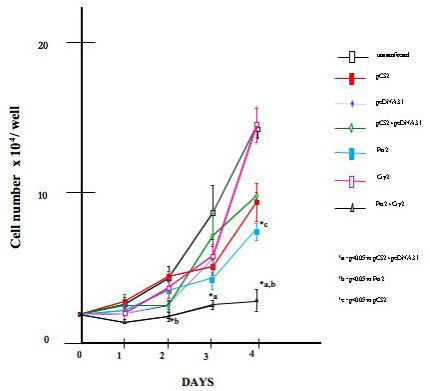
**Effect of *PER 2 *and *CRY 2 *on the growth of MCF-7 cells**. Cell proliferation assays were conducted on parental, vector-transfected, *PER 2 *overexpressing MCF-7 cells, *CRY 2 *overexpressing MCF-7 cells, and cells over expressing both *PER 2 *and *CRY 2*. Cells were counted on a hemacytometer using the trypan blue stain every day for four days. N = 3 independent experiments in triplicate. *a = p < 0.05 vs. pCS2+pCDNA3.1, *b = p < 0.05 vs. Per2, *c = p < 0.05 vs. pCS2

Cell proliferation assays were conducted on parental, vector-transfected, *Per 2 *transfected and expressing MCF-7 cells, *Cry 2 *transfected and expressing MCF-7 cells, and MCF-7 cells transfected with and expressing both *Per 2 *and *Cry 2*. Following transfection, cell number and cell viability were assessed by hemacytometer cell counts and trypan blue staining (Figure 2). A significant 31.6% decrease in cell number was seen in cells expressing *PER 2 *on day 4, but not in cells expressing *CRY 2*. However, when both *PER 2 *and *CRY 2 *were expressed in the same cells a significant 44.6% and 61.81% suppression of cell proliferation was noted as compared to control cells on day 2 and day 4 respectively.

### PER 2 expression reduces MCF-7 cell growth in soft agar

**Figure 3:**
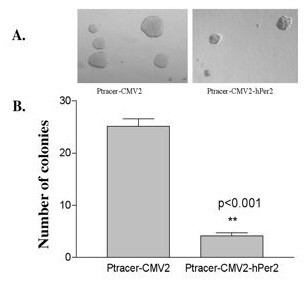
***PER 2 *expression reduces MCF-7 cell growth in soft agar**. Soft agar clonogenic assays were performed with MCF-7 cells. MCF-7 cell were transfected with pTracer™-CMV2 or pTracer™-CMV2-hPer 2 and sorted. Two thousand five hundred cells per well were seeded in 12-well plates (3.8 cm^2^) in culture medium containing 0.35% low-melting agarose over a 0.7% agarose base layer and incubated for 12 days. Colonies larger than 100 μm in diameter were counted.

To assess the effect of *PER 2 *on *in vitro *tumorigenicity, soft agar clonogenic assays were performed with MCF-7 cells (Figure 3). After 12 days in culture, control cells transfected with the pTracer™-CMV2 displayed a clonogenic efficiency of 27% (25.00 ± 1.86 colonies), whereas MCF-7 cells transfected with and expressing *PER 2 *demonstrated a significantly reduced efficiency to 1.6% (4.11 ± 0.86 colonies). The difference in clonogenic efficiency between these two cell groups was determined to be highly significant (P < 0.001). In addition to clonogenic efficiency, the size of colonies formed by pTracer™-CMV2 vector transfected cells (376 ± 37 μm) was significantly larger (*p *< 0.05) than those formed by *Per 2 *transfected cells (163 ± 18 μm).

### Effect of PER 2 and CRY 2 on the cell cycle of MCF-7 cells

To determine if the decrease in cell proliferation induced by the expression of *PER 2 *or *PER 2 *and *CRY 2 *is a result of alteration in the cell cycle, we conducted cell cycle analyses on parental, vector-transfected, *Per 2 *transfected and expressing MCF-7 cells, *Cry 2 *transfected and expressing MCF-7 cells, and cells transfected with and expressing both *Per 2 *and *Cry 2 *(Table 1). The expression of *PER 2 *in MCF-7 cells resulted in a significant increases (25.8%) in the percentage of cells in the G1 phase of the cell cycle as compared to vector transfected controls and a significant decrease (60%) in the percentage of cells in S phase, vs. vector transfected controls. The combined expression of both *PER 2 *and *CRY 2 *resulted in an even greater increase (42.5%) in the percentage of cells in G1 by demonstrating a blockade of MCF-7 breast cancer cells blocked at the G1/S border.

**Table 1:** Effect of *PER 2 *and *CRY 2 *on the cell cycle of MCF-7 cells

	G1	S	G2/M
GFP	7414	15.57	10.29
pCS2	65.36	18.03	16.61
PER2	82.24	7.16	10.60
pCS2 + pcDNA3.1	62.61	17.49	19.90
PER2 + CRY2	89.19	0.58	10.22

Cell cycle analyses were conducted on parental, vector-transfected, *PER 2 *overexpressing MCF-7 cells, *CRY 2 *overexpressing MCF-7 cells, and cells over expressing both *PER 2 *and *CRY 2*. After three days of expression, propidium iodide(PI) staining and samples were analyzed by flow cytometry.

### PER 2 expression induces apoptosis in MCF-7 breast cancer cells

**Figure 4:**
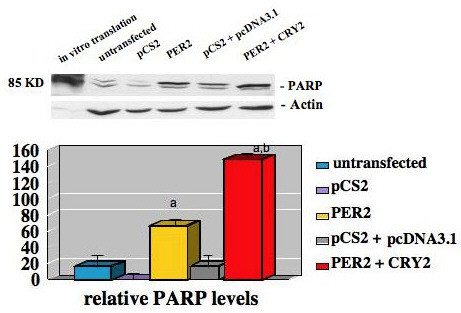
***PER 2 *expression induces apoptosis in MCF-7 breast cancer cells**. **(A) **MCF-7 cells were transiently transfected with empty vector, vector expressing *PER 2*, or both vectors expressing *PER 2 *or *CRY 2*. After 72 hours, total cell extracts were prepared and subjected to Western blot analysis using an antibody directed against the 85 kDa cleaved fragment of PARP [poly (ADP-ribose) polymerase] (representative of three independent studies). **(B) **Densitometric analysis of cleaved PARP protein (average value of three independent studies). a. *PER 2 *or *PER 2 *+ *CRY 2 *vs vector control, p < 0.001, b *PER 2 *+ *CRY 2 *vs *PER 2*, p < 0.01

To determine if the decreased growth in MCF-7 cells transfected with *Per 2 *or *Per 2 *and *Cry 2 *was the result of increased apoptosis we conduced PARP-cleavage assays. Seventy two hours post transfection cells were harvested and total cell extracts prepared and subjected to Western blot analysis using an antibody directed against the 85 kDa cleaved fragment of PARP [poly (ADP-ribose) polymerase] (Figure 4). MCF-7 cells transfected and expressing *Per 2 *displayed a significant increase (*p *< 0.001) in cleaved PARP protein levels by 714.2% when compared to control vector transfected cells. These results show that expression of *PER 2 *induces apoptosis in MCF-7 human breast cancer cells and that concomitant expression of *PER 2 *and *CRY 2 *further increase apoptosis in MCF-7 human breast cancer cells by 689.5% (*p *< 0.001) compared to transfection controls and 74% (*p *< 0.01) compared to cells expressing *PER 2 *alone.

### Effect of PER 2 on the expression of P53 and Cyclin D1

**Figure 5:**
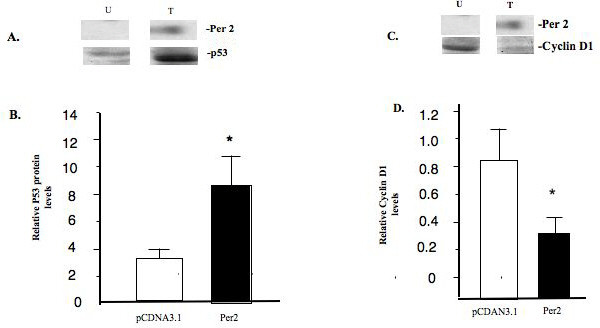
***PER 2 *expression increases P53 but decreases cyclin D1 expression in MCF-7 cells**. **(A) **MCF-7 cells were transiently transfected with and empty vector (pcDNA 3.1) [U] or vector expressing *PER 2 *[T]. After 48 hours, total cell extracts were prepared and subjected to Western blot analysis using an antibodies directed against the P53 and *PER 2 *proteins. **(B) **Densitometric analysis of P53 protein expression. * p < 0.05. **(C) **Western blot analysis using an antibodies directed against the cyclin D1(cD1) and *PER 2 *proteins. **(D) **Densitometric analysis of cD1 protein expression. * p < 0.05.

Given that expression of *PER 2 *and *PER 2 *plus *CRY 2 *in MCF-7 cells results in decreased cell proliferation, alters the cell cycle, and induces apoptosis, we asked if there were associated changes in the expression of the cyclin D1 cell cycle associated gene and the DNA repair/apoptosis associated p53 protein. Seventy two hours following transfection with an empty vector (pcDNA 3.1) or the *Per 2 *expression construct, total cellular protein was extracted, prepared and subjected to Western blot analysis using antibodies directed against human p53 and cyclin D1 (Figure 5). Compared to vector transfected control cells, *PER 2 *expressing cells displayed significantly elevated levels (157% of controls) of p53 protein but significantly decreased levels (56% reduced vs. controls) of cyclin D1 protein.

## Discussion

Substantial epidemiological and clinical data demonstrate that circadian rhythms greatly affect the diagnosis and outcome of breast cancer patients. However, the chronotherapeutic observations leading to the current standard of treatment are serendipitous and lack a strong cellular and molecular rationale. In the present study, we provide further evidence indicating a role of circadian rhythms and regulation of the cellular clock in the control of the cell cycle and in human breast cancer cell growth and proliferation. A common molecular circuitry is seen in both central and peripheral oscillators [3,4]. The clockwork machinery comprises a battery of transcriptional activators and repressors that form an auto-regulatory transcriptional feedback loop. Clock and BMAL1 are paired transcriptional activators that drive the expression of *PER 1, 2, 3 *and *CRY 1, 2 *and the nuclear orphan receptor gene Rev-erbα. The *PER/CRY *protein complex inhibits the transcription of its own genes by the Clock/BMAL1 transcriptional complex, while Rev-erbα binds to ROREs in the BMAL1 promoter to block BMAL1 expression [1,2,29,30]. This cell-autonomous feedback loop permits cyclic expression of oscillator genes at various phases with the same periodicity of approximately 24 h [2,31].

As a core clock gene, *Per 2 *functions to maintain the circadian rhythm of the SCN and peripheral cells and also to sustain the normal cell cycle. In our studies, contrary to the report of Gery et al. [32], we found that *PER 2 *was expressed in normal breast epithelial cell lines (HMCE-Tert and MCF-10A) and in the ERα-negative MDA-MB-231 breast tumor cell line, but not in MCF-7 and T47D breast cancer cell lines. Several possibilities exist for this discrepancy including the possibility that, in light of the report that acute treatment of MCF-7 cells with estrogen can modulate *PER 2 *expression, long term exposure to E2 in our culture media may down regulates *PER 2 *expression. It is also possible, that our MCF-7 line demonstrates a different gene expression pattern than those used by Gery et al. [32].

Our studies do, however, confirm that, when *PER 2 *is expressed at elevated level in MCF-7 breast cancer cells, it induces a significant growth-inhibitory effect. When *PER 2 *and *CRY 2 *are co-expressed an even greater enhancement of growth-inhibition was seen as compared to cells expressing only *PER 2*. Interestingly, the expression of *CRY 2 *alone had no significant effect on MCF-7 cell proliferation, suggesting *CRY 2 *is itself not a critical regulator of cell cycle but that it functions to regulate cell cycle and cell death in breast cancer cells by partnering with *PER 2*. This inhibition of cell proliferation by *PER 2 *and *PER 2/CRY2 *may be related to several events, including alteration of the cell cycle and induction of apoptosis. As shown in our studies, expression of *PER 2 *alone, or *PER 2/CRY 2 *blocked MCF-7 cell cycle at the G1/S border. The anti-proliferative effects of *PER 2 *appear to be attributable to the induction of apoptosis rather than just an elongation or blockade of the cell cycle, as *PER 2 *expression in MCF-7 cells induces morphological changes, such as rounding up and detachment that are consistent with programmed cell death. The induction of apoptosis by *PER 2 *was confirmed by a significant increase in PARP [poly(ADP-ribose)polymerase] cleavage, an indicator of activated caspases [33].

Recent studies have reported that clock-controlled genes are involved in the regulation of the cell cycle and apoptosis, including c-*Myc*, the tumor suppressor *p53*, and cyclins [28]. Progression through G1 depends initially on cyclin D-CDK4/6 protein complexes, and later on cyclin E-CDK2. Cyclin D1 is a key cell cycle regulatory protein, known to be up-regulated by estrogen, an established breast cancer mitogen [34]. The down-regulation of cyclin D1 plays an important role in the cell cycle arrest. Our results indicate that expression of *PER 2 *significantly down-regulates cyclin D1 level in MCF-7 cells, which may contribute to the arrest in G1 of the cell cycle. Loss of p53 in many cancers leads to impaired cell cycle regulation, genomic instability and inhibition of apoptosis. The tumor suppressor p53 can induce a transient arrest in G1 in cells, allowing cells time to repair damaged DNA [35]. Activated p53 can also eliminate cells through mechanisms involving prolonged arrest in G1 and induction of apoptosis. The elimination of abnormally proliferating cells by p53 is considered to be the principal means by which p53 mediates tumor suppression [35,36]. The expression of *PER 2 *in MCF-7 cells significantly increase p53 levels. Our data confirms in human breast cancer cells the results obtained by Hua et al [36] in *Per 2 *mutant mice. The elevated expression of P53 in *PER 2 *expressing breast cancer cells may contribute, at least in part, to both arrest in G1 of the cell cycle and apoptosis. *PER 2 *may induce tumor cell apoptosis by the p53-mediated mitochondrial signaling pathway. The mechanism by which *PER 2 *regulates Cyclin D1 and p53 expression is under investigation in our laboratory.

Anchorage-independent growth in soft agar as a characteristic of *in vitro *tumorigenicity is correlated with enhanced tumor progression and metastasis and is a hallmark of malignant transformation and, thus, is an effective method to evaluate the growth and tumorigenicity of cells *in vitro *[37-39]. Our studies demonstrate that expression of *PER 2 *results in a significant decrease in the number and size of colonies formed by MCF-7 cells in soft agar. Together these data demonstrate that expression of *PER 2 *significantly inhibits anchorage-independent growth of MCF-7 cells.

Finally, Western blot analysis demonstrates that *PER 2 *is endogenously expressed in normal/transformed human breast epithelial cell lines but is not expressed or expressed at a significantly reduced level in human breast cancer cell lines. The fact that expression of *PER 2 *alone inhibits MCF-7 cell proliferation and induces apoptosis suggests that reduced expression or altered function of *PER 2 *may be a mechanism via which mammary epithelial cells escape programmed cell death and progress to a malignant phenotype.

Our studies, as well as those of Fu et al. [28] and Gery et al. [32] clearly demonstrate the tumor suppressive nature of *PER 2 *as evinced by inhibition of cell growth, induction of apoptosis, reduced colony formation and growth in soft agar. Furthermore, our studies shown that, although *PER 2 *can function independently as a tumor suppressor, its activity is significantly enhanced in the presence of its normal clock partner *CRY 2*. Based on our data and on results previously described in the literature, we feel confident that the loss of *PER 2 *is associated with some forms of breast cancer. However, we cannot say for certain if the link between loss of *PER 2 *function and cancer development is mediated by disruption of circadian rhythmicity. Numerous studies have demonstrated that clock genes, including *Per 2*, are expressed in peripheral tissues, and that some are subservient to the master clock in the SCN. Future studies will address the association and regulation of the SCN clock by photoperiod and the feed down effect of photoperiod onto clock function in peripheral cells including the epithelium of t he breast.

## Abbreviations

*Per 2*: Period 2, *Cry 2*: Cryptochrome 2, PARP: poly (ADP-ribose) polymerase BMAL1: Brain-muscle-arnt-like 1

## Authors' contributions

SX conducted experimental work on individuals, and primarily developed the manuscript. SBC participated in cell growth study and western blots for p53 and cyclin D1. LLM participated the western blots for *PER 2*. LY and QC participated in cell culture. SMH is the PI of project and secured the DOD and NIH/NCI grant under which this research was funded. All authors read and approved the final manuscript.

## References

[B1] Richter HG, Torres-Farfan C, Rojas-Garcia PP, Campino C, Torrealba F, Seron-Ferre M (2004). The circadian timing system: making sense of day/night gene expression. Biol Res.

[B2] Reppert SM, Weaver DR (2002). Coordination of circadian timing in mammals. Nature.

[B3] Albrecht U, Eichele G (2003). The mammalian circadian clock. Curr Opin Genet Dev.

[B4] Reppert SM, Weaver DR (2001). Molecular analysis of mammalian circadian rhythms. Annu Rev Physiol.

[B5] Welsh DK, Logothetis DE, Meister M, Reppert SM (1995). Individual neurons dissociated from rat suprachiasmatic nucleus express independently phased circadian firing rhythms. Neuron.

[B6] Liu C, Weaver DR, Strogatz SH, Reppert SM (1997). Cellular construction of a circadian clock: period determination in the suprachiasmatic nuclei. Cell.

[B7] King DP, Zhao Y, Sangoram AM, Wilsbacher LD, Tanaka M, Antoch MP, Steeves TD, Vitaterna MH, Kornhauser JM, Lowrey PL, Turek FW, Takahashi JS (1997). Positional cloning of the mouse circadian clock gene. Cell.

[B8] Darlington TK, Wager-Smith K, Ceriani MF, Staknis D, Gekakis N, Steeves TD, Weitz CJ, Takahashi JS, Kay SA (1998). Closing the circadian loop: CLOCK-induced transcription of its own inhibitors per and tim. Science.

[B9] Gekakis N, Staknis D, Nguyen HB, Davis FC, Wilsbacher LD, King DP, Takahashi JS, Weitz CJ (1998). Role of the CLOCK protein in the mammalian circadian mechanism. Science.

[B10] Jin X, Shearman LP, Weaver DR, Zylka MJ, de Vries GJ, Reppert SM (1999). A molecular mechanism regulating rhythmic output from the suprachiasmatic circadian clock. Cell.

[B11] Kume K, Zylka MJ, Sriram S, Shearman LP, Weaver DR, Jin X, Maywood ES, Hastings MH, Reppert SM (1999). mCRY1 and mCRY2 are essential components of the negative limb of the circadian clock feedback loop. Cell.

[B12] Shearman LP, Jin X, Lee C, Reppert SM, Weaver DR (2000). Targeted disruption of the mPer3 gene: subtle effects on circadian clock function. Mol Cell Biol.

[B13] Zhou YD, Barnard M, Tian H, Li X, Ring HZ, Francke U, Shelton J, Richardson J, Russell DW, McKnight SL (1997). Molecular characterization of two mammalian bHLH-PAS domain proteins selectively expressed in the central nervous system. Proc Natl Acad Sci U S A.

[B14] Balsalobre A, Damiola F, Schibler U (1998). A serum shock induces circadian gene expression in mammalian tissue culture cells. Cell.

[B15] Filipski E, Li X, Levi F (2006). Disruption of circadian coordination and malignant growth. Cancer Causes & Control.

[B16] Filipski E, King VM, Li XM, Granda TG, Mormont MC, Claustrat B, Hastings MH, Levi F (2003). Disruption of circadian coordination accelerates malignant growth in mice. Pathologie Biologie.

[B17] Stevens RG, Rea MS (2001). Light in the built environment: potential role of circadian disruption in endocrine disruption and breast cancer. Cancer Causes Control.

[B18] Hansen J (2001). Increased breast cancer risk among women who work predominantly at night. Epidemiology.

[B19] Patel D (2006). Shift work, light at night and risk of breast cancer. Occup Med (Lond).

[B20] Davis S, Mirick DK (2006). Circadian disruption, shift work and the risk of cancer: a summary of the evidence and studies in Seattle. Cancer Causes Control.

[B21] Stevens RG (2005). Circadian disruption and breast cancer: from melatonin to clock genes. Epidemiology.

[B22] Davis S, Mirick DK, Stevens RG (2001). Night shift work, light at night, and risk of breast cancer. J Natl Cancer Inst.

[B23] Schernhammer ES, Laden F, Speizer FE, Willett WC, Hunter DJ, Kawachi I, Colditz GA (2001). Rotating night shifts and risk of breast cancer in women participating in the nurses' health study. J Natl Cancer Inst.

[B24] Hill SM, Spriggs LL, Simon MA, Muraoka H, Blask DE (1992). The growth inhibitory action of melatonin on human breast cancer cells is linked to the estrogen response system. Cancer Lett.

[B25] Hill SM, Blask DE (1988). Effects of the pineal hormone melatonin on the proliferation and morphological characteristics of human breast cancer cells (MCF-7) in culture. Cancer Res.

[B26] Filipski E, King VM, Li XM, Granda TG, Mormont MC, Liu XH, Claustrat B, Hastings MH, Levi F (2002). Host circadian clock as a control point in tumor progression. Journal of the National Cancer Institute.

[B27] Bartsch H, Bartsch C (1981). Effect of melatonin on experimental tumors under different photoperiods and times of administration. J Neural Transm.

[B28] Fu LN, Pelicano H, Liu JS, Huang P, Lee CC (2002). The circadian gene Period2 plays an important role in tumor suppression and DNA damage response in vivo. Cell.

[B29] Gallego M, Virshup DM (2007). Post-translational modifications regulate the ticking of the circadian clock. Nat Rev Mol Cell Biol.

[B30] Shearman LP, Sriram S, Weaver DR, Maywood ES, Chaves I, Zheng B, Kume K, Lee CC, van der Horst GT, Hastings MH, Reppert SM (2000). Interacting molecular loops in the mammalian circadian clock. Science.

[B31] King DP, Takahashi JS (2000). Molecular genetics of circadian rhythms in mammals. Annu Rev Neurosci.

[B32] Gery S, Virk RK, Chumakov K, Yu A, Koeffler HP (2007). The clock gene Per2 links the circadian system to the estrogen receptor. Oncogene.

[B33] Boulares AH, Yakovlev AG, Ivanova V, Stoica BA, Wang G, Iyer S, Smulson M (1999). Role of poly(ADP-ribose) polymerase (PARP) cleavage in apoptosis. Caspase 3-resistant PARP mutant increases rates of apoptosis in transfected cells. J Biol Chem.

[B34] Castro-Rivera E, Samudio I, Safe S (2001). Estrogen regulation of cyclin D1 gene expression in ZR-75 breast cancer cells involves multiple enhancer elements. J Biol Chem.

[B35] Benchimol S (2001). p53-dependent pathways of apoptosis. Cell Death Differ.

[B36] Hua H, Wang YQ, Wan CM, Liu YY, Zhu B, Yang CL, Wang XJ, Wang ZR, Cornelissen-Guillaume G, Halberg F (2006). Circadian gene mPer2 overexpression induces cancer cell apoptosis. Cancer Science.

[B37] Nikiforov MA, Hagen K, Ossovskaya VS, Connor TM, Lowe SW, Deichman GI, Gudkov AV (1996). p53 modulation of anchorage independent growth and experimental metastasis. Oncogene.

[B38] Clezardin P (1998). Recent insights into the role of integrins in cancer metastasis. Cell Mol Life Sci.

[B39] Nakanishi K, Sakamoto M, Yasuda J, Takamura M, Fujita N, Tsuruo T, Todo S, Hirohashi S (2002). Critical involvement of the phosphatidylinositol 3-kinase/Akt pathway in anchorage-independent growth and hematogeneous intrahepatic metastasis of liver cancer. Cancer Res.

